# Sustainability pedagogy: Understanding, exploring and internalizing nature’s complexity and coherence

**DOI:** 10.3389/fpsyg.2022.922275

**Published:** 2023-01-04

**Authors:** Nicole Spiegelaar

**Affiliations:** Trinity College and School of the Environment, University of Toronto, Toronto, ON, Canada

**Keywords:** sustainability pedagogy, Restoration Theory, directed attention, complexity, resilience, pandemic, nature immersion, agency

## Abstract

Online learning during the COVID-19 pandemic has affected student academic performance as well as mental, physical, and social wellbeing. During a lockdown at the University of Toronto in Canada (September 2020–April 2021), my students expressed an underlying sense of *monotony* yet *uncertainty*. I recalled a contrasting paradox from the teachings of Indigenous Cree on mental wellness in land-based experiences: a sense of *stimulation* and *security* that we can liken to variations of Appleton’s prospect-refuge theory. I modified my *Environmental Science and Pathways to Sustainability* course to support stimulation and security through embodied, interactive pedagogy at student-selected individual field sites. My main goals were to (i) support student mental wellness and (ii) provide an alternative to experiential field trips for understanding and connecting with nature as an adaptive complex system. I prompted students with field activities contextualized by a course narrative that purposefully directed attention to nature through intrinsically motivated curiosity, exploration, and discovery; conditions more similar to evolutionary environments of adaptedness than “getting away” in passive retreats. Student weekly field observations and reflections culminated in a post-intervention Reflection Assignment (*n* = 15) which became the bases of thematic and narrative analysis. Other assignments were added to my evaluation of complexity comprehension. The intervention successfully instilled security and stimulation *via* purpose-directed attention to different aspects of nature in the same setting followed by periods of knowledge integration. This empowered students with *sustainability mindsets* indicated by greater self-reported: sense of coherence, change agency, cognitive and affective restoration, nature connectedness, nature relatedness, social connectedness, and pro-environmental values. Assignments demonstrated an understanding of the environment as an adaptive complex system that was not present at the beginning of the course. Some students’ self-construct adopted nature and its complexity, empowering them with greater trait resilience. This work speaks to opportunities for merging psychological restoration and analytical curricula by integrating cognitive and sensory meaningfulness in sustainability narratives. It asks scholars to reflect on how we operationalize foundational theories of Environmental Psychology based on ancestral survival conditions and encourages empirical research to consider how sociocultural contexts can direct attention to nature through purposeful inquiry.

## Introduction

During the COVID-19 pandemic, university students experienced a deterioration in wellbeing correlating with lockdown severity (Rogowska et al., 2020; Wang et al., 2020; Szczepańska and Pietrzyka, 2021). Among US university students, less screen time (<8 h/d) and more outdoor time (≥2 h/d) buffered against numerous negative psychological impacts of COVID, including stress and preoccupation with the virus (Browning et al., 2021). In response to restrictions on social gathering, university instructors have been nudged to move the classroom outdoors, simultaneously taking advantage of the known nature-immersion benefits on student learning and wellbeing (Ayotte-Beaudet et al., 2020; Brookfield, 2021).

Outdoor pedagogies are very common in child and youth education, as in ecoschools, open schools, forest schools, or the Danish “Udeskole” (Bentsen et al., 2018). While outdoor education is gaining importance at the university level with sustainability priorities, it remains relatively uncommon due to a history of being physically and conceptually separate from “real” curriculum (Brookes, 1989; Lugg, 2007). We still tend to see more focus on non-academic recreation and leisure as restorative getaways from real work (Andre et al., 2017; Davidson and Ewert, 2020). While university environmental sciences often provide abundant outdoor learning opportunities, they tend to focus on “hard” analytical skills and the pedagogical research lacks attention to the cognitive, affective, and social dynamics that take place. Thus, we see a gap between university pedagogies, outdoor education, and Environmental Psychology.

This separation of nature-based activities from daily routine is a product of the post-industrial modern period. Blühdorn (2016) aptly summarizes how the modern individual has become alienated from social and ecological relationships once fostered in collectivist, land-based societies, is more subject to accelerated change (information, transportation, virtualization), and is thus more individualized, extended, and differentiated; further, that this is inextricably linked to our present socio-ecological crises. Along with Payne and Wattchow (2009), I believe this is reinforced in conventional higher education, where learning is a disembodied experience of siloed specialization, isolated from corporal and interconnected realities. These limitations are compounded with social isolation and virtual dependence of pandemic lockdowns.

### Sustainability mindsets

How do we prepare environment and sustainability students with the personal and professional capacity to address human-nature devastation on Earth? As people who are deeply aware of this crisis and engaged in trying to solve it, these students are at greater risk of psychological distress and becoming paralyzed by anxiety, despair, and powerlessness (Fraser et al., 2013; Wallace et al., 2020).

Nature-immersion pedagogies can support these students by empowering them with applied field skills and mutually reinforcing traits of nature connectedness, psychological wellbeing, and pro-environmental values (Nisbet et al., 2011; Redondo et al., 2021). Yet necessary change cannot be addressed by simply caring about or measuring the environment; sustainability challenges must be approached with multiple worldviews in place-based, sociocultural contexts, and require interdisciplinarity literacy (Dale and Newman, 2005; Loring, 2020). Outdoor sustainability education is formative in traits of social conscientiousness, self-esteem, self-discipline (Torkos, 2017), and change agency (Irwin, 2010) that are necessary for organizational change. Sandell and Öhman (2010) specify that nature must be experienced in relation to ethical and sociocultural contexts and understood as a dynamic, process-oriented concept.

This concept, known as adaptive complex systems, is promoted as a theoretical framework supporting mutual human-nature *thriving* (Du Plessis and Brandon, 2015; Loring, 2020). Davis et al. (2018) define an adaptive complex system as a dynamic whole that is more than the sum of interdependent parts. Their research has demonstrated that university students who are systems thinkers have stronger understanding of complex ecological systems and their influence on socioeconomic domains; nature connectedness; social empathy; pro-environmental values and behaviors; and tolerance for ambiguity (Trovarello and Stroink, 2015; Davis and Stroink, 2016; Davis et al., 2018). Training students to think in terms of complexity alone may be a powerful tool for developing sustainability mindsets, and responding to uncertainty, stress, and isolation.

A critical aspect of human psychological *thriving* is a sense of coherence—the *enduring* yet *dynamic* sense that stimuli of both the *internal and external* environment have the three dimensions of comprehensibility, manageability, and meaningfulness (Antonovsky, 1996; Vinje et al., 2017). Consider the impact of ecoanxieties about distal and often intangible phenomenon beyond our individual control. Our understanding of sense of coherence can play an important role in educating healthy and impactful sustainability leaders, yet research at this intersection has not been explored.

### Purpose-directed attention

Decades of research in Environmental Psychology has supported the theory that nature can restore our cognitive and affective capacities after they are depleted by urban stressors. This work is built upon an underlying Evolutionary Psychology theory that our ancestral psycho-physiological systems evolved to handle survival challenges of natural settings in our *Evolutionary Environment of Adaptedness* (EEA; Tooby and Cosmides, 1990): relatively foreign modern environments can present a discord or mismatch unsuitable for our mind-bodies can be relieved by nature experiences (Kaplan and Kaplan, 1989: 73, 95; Joye, 2007; Grinde and Patil, 2009).

The mechanism of affective restoration offered by the *Stress Recovery Theory* of Ulrich et al. (1991) is based on automatic and unconscious attention to nature’s beauty as an escape from daily demands. The Kaplans’ *Attention Restoration Theory* proposes that modern settings deplete attentional stores through *forced*, *directed* attention on *mundane* tasks; cognitive restoration occurs in nature because it has the attributes of being safe, interesting, and “away from daily demands,” inspiring a *soft, effortless fascination* and undirected (involuntary) attention (Kaplan and Kaplan, 1989; Kaplan, 1995).^1^ However, these explanations ignore the fact our EEA did not separate nature from daily routine. Dualities of urban-nature, human-wilderness, and recreation-routine are modern constructs. Others have recently argued that land-based survival obviously demands ongoing, effortful attention and hard fascination (Joye and Dewitte, 2018).

Indigenous Cree harvesters (James Bay, Canada) shared with me that land-based survival requires constant, careful attention to changing patterns, symbols, and relationships in order to predict survival challenges and opportunities (*in progress*); moreover, survival is navigated through shared sociocultural meaning and a nature-based identity (Adelson, 2000; Grey and Patel, 2015). As such, Cree expressed that attention to these challenges, patterns, and relationships is intrinsically motivated (not forced) and enjoyable. I thus consider how to emulate this purposeful, semi-directed attention style in nature-based pedagogies.

### Security and stimulation

I propose that nature settings offer cognitive and affective benefits insomuch as they provide security and stimulation. Again, I was inspired by Cree land-based practices, where the need to be prepared drives exploration and the joy of meaningful discovery. Understanding the environment fosters a sense of intimacy, trust, and safety, which helps cope with change and uncertainty.

This idea that the human psyche is enriched by natural settings with a balance of security and stimulation is not new. It is also presented in Appleton’s (1975) theory based on *prospect,* potential to obtain more information, and *refuge,* concealment for safety (Appleton, 1984), and Preference Matrix of Kaplan (1995) based on *understanding* and *exploring*: more specifically, desirable natural environments offer *coherence* and *legibility—*organized relationships and points of orientation—as well as *complexity* and *mystery—*opportunities for discovery.

However, I propose that when greater meaning is derived from nature (as in collective survival), security and stimulation permit and promote the purpose-directed attention described above. Further, this drives a cycle of curiosity, exploration, and discovery that generates more meaning and intimacy with the environment, and reduces attentional effort, thus reinforcing positive feedback toward purpose-directed attention, security and stimulation.

In a virtual, fast-paced world of monotonous uncertainty, detached from the social and natural relations of production that support our own survival, we have few reasons to be attentive to subtleties in nature. My pedagogical approach attempted to create purpose-directed attention to nature using field activities and supporting class narratives with the aim of drawing attention to nature’s complexities while promoting sense of stability and joyful discovery.

### Theoretical framework

I have the following working theories which informed my pedagogical approach:

- Interactive, embodied nature observation can harness our innate evolutionary need to derive psycho-social benefits from purposeful attention to nature; this is enhanced by integration periods of reflection and shared social experiences
- (Semi-) Directed attention in nature is intrinsically motivated when the information uncovered through discovery can be applied to a shared sociocultural narrative
- Revisiting a natural setting over time strengthens connection and comprehension of nature; along with gradual introduction of new information (changes in nature, exploration, sociocultural problem solving), it fosters a balance of security and stimulation that supports psychological thriving, this drives a cycle of further curiosity, exploration, discovery, and intimacy with the space
- Thus, sense of security/stimulation and purposeful (semi-)directed attention are mutually reinforced; focused and sustained attention is more restorative as trust and discovery increase
- Sense of coherence with internal and external realities is strengthened through joint attention to meaningful sensory-perceptual-conceptual realities in a given moment; this reinforces the security/stimulation cycle above with greater agency and awareness

I did not empirically test these interacting theories in this retroactive study of secondary qualitative data. By presenting these theories congruently along with student testimony excerpts (Results), I hope to influence experimental design and emerging theory development in Environmental Psychology.

### The case study

The intervention took place in a small first-year university *Environmental Science and Sustainability* course that prepares students for diverse programs of study across the humanities, social sciences, and physical sciences.^2^

My broader learning objectives when designing the course in 2018 were for students to develop (i) an understanding of, and connection to, the natural environment, (ii) a sustainability lens based on adaptive complex systems that they can apply to any discipline, and (iii) basic literacy, communication, analysis, and problem solving in environmental science situated in sociocultural contexts of sustainability. Prior to the pandemic, I used experiential pedagogy *via* field trips to natural areas, an ecological research station, campus garden spaces, farms, and sustainability organizations.

According to my students, these applied settings helped “non-science” majors better understand science and “science” majors better connect biophysical elements to subjective sociocultural contexts. Interdisciplinary majors were better able to see connections between disciplines and expressed reduced anxiety about taking on a program of study that carries more uncertainty, is less specialized, and does not have a defined career outcome.

The pandemic lockdown from September 2020 to April 2021 restricted this method. Moreover, students expressed feeling isolated, hopeless, and both a sense of anxiety due to uncertainty, as well as a sense of monotony due to lack of stimulation. Rather than reducing course work, I was inspired to change the qualitative features of student learning experiences in the form of the intervention.

### Intervention objectives

I employed an outdoor intervention with the pedagogical goal of supporting student sustainability mindsets, including the following outcomes:

- Standard course goals:
  - Introductory environmental science literacy and skills through experiential learning: comprehension, analysis, field observations, and communication
  - Embodied understanding of, and connection to, nature as a complex system
  - Understanding and application of adaptive complex systems of sustainability
- Mental wellness, social connectedness, and sense of security/stimulation during lockdown uncertainty, monotony, and isolation
- Sense of coherence—internal and external environments feel comprehensible, manageable, and meaningful—related to personal and environmental awareness and agency

I sought to support these outcomes by applying my Theoretical Framework above. I attempted to intrinsically motivate embodied and purpose-directed attention to novel entities and relationships in the same natural setting *via* meaningful course narratives (readings, discussion, field prompts, and online posts) that promote a cycle of curiosity, exploration, discovery, and nature connectedness, and support knowledge integration.

## Methodological approach

The overall approach involved a nature-based intervention, weekly journal entries, and a final Reflection Assignment on participant experience that was used for data analysis. This Assignment, along with standard course assignments, was used to evaluate complexity comprehension and environmental perceptions. During the course, the students were not aware of my intervention strategy or the theory that informed it.

To develop the intervention, I applied the above theories in response to student needs, seasons, assignments, and coarse goals. Here, I share my broad strategies and provide select examples, but encourage readers to use their own variations of these strategies to best suit unique student populations, course goals, and disciplinary knowledge.

### Weekly activities

Students chose a natural setting and a tree that would be accessible all year. For Weeks 2–8 of their first semester, they were instructed to:

- Complete readings on the weekly topic
- Conduct independent research that applies the topic to their setting or tree
- Follow “prompts” for field activities with their nature setting and/or tree
- Document observations in a field book (optional: personal journaling)
- Post photos of features that reflect the weekly topic (online discussion forum)
- Post key insights and answers to questions based on their readings, research, and observations (online discussion forum)
- Engage in seminar discussions about findings and experiences of field activities in the context of weekly topics

We explored topics such as ecology, evolution, conservation, invasive species, plant identification, phenology, hydrology, citizen science, soils, and anthropogenic change.

### Strategies

Weekly activities permitted basic experiential learning and environmental analyses otherwise limited by the lockdown. I designed them with careful placement of continuity and novelty (security and stimulation) to simultaneously reduce lockdown distress, requiring no additional time commitment from students. Security and stimulation were fostered through new concepts or questions applied to the same space, or by returning to the same questions/concepts with different perspectives, in different seasons, or in an urban setting.

Weekly activity prompts and discussion questions required students to (i) discover their setting and tree to find answers, and in the process (ii) apply the course narrative (readings, independent research, and discussion questions) to their setting/tree, and vice versa. The meaning derived through this feedback over time was intended to foster greater connection to place and purpose-directed attention to novel entities and relationships in that place. I designed questions to intrinsically motivate discovery with their unique setting and tree, supporting skills in environmental science inquiry. Meaning, students were not given replicable, step-by-step instructions of what to look at; they were taught how to look for information and given questions that required careful observation (semi-directed attention). Application of field observations to class discussions and assignments gave students an opportunity to apply environmental science facts to place-based sociocultural contexts, seeing differences among their peers in unique ecological, geographical, and international settings.

Opportunities for social field work and shared navigation were extremely limited. Students connected over similar field experiences through the online discussion forum and online seminar. Posts of photos and findings were also intended to support social connection by sharing experiences through a familiar practice (i.e., social media).

I diversified the scope of attention and levels of engagement to introduce novelty and embodied interactions. I included more interactive tasks (e.g., digging and analyzing soil) and observations from different perspectives over time (e.g., factors in the environment that contribute to soil type), with different sensory experiences. Questions directed student attention to patterns of detailed features (e.g., leaf morphology) and broader systems (e.g., phenological change), as well as the relationships between features (e.g., mutualism and competition, invasive species traits, and root-fungal networks). My goals here were to encourage complex systems thinking by pivoting between reductionist and holistic scope of attention, thereby training mindsets that can place scientific facts in sociocultural contexts and overcome disciplinary conditioning in “science versus non-science” mindsets. To this end, I mixed analytical, creative, and reflection tasks, and had students consider this knowledge in immediate and distal contexts.

This practice was also intended to support their ability to see how life works on multiple scales, and to understand the ecological patterns of adaptive complex systems. Students were prompted to think of patterns of change based on interactions of features in the past, present, and future, as a place-based exercise in understanding anthropogenic change.

In Week 1, they were asked to write a definition of *environment*. By Week 2, they carefully observed their setting and tree, documenting the entities and interactions in this setting, and again defined *environment*. This was intended to contrast embodied and disembodied conceptions, to prime students for field observation, and to provide baseline notes of their perception, state, and beliefs while in their natural setting before diving into the material. Unlike past years, students were encouraged to use field books to free write about personal thoughts, feelings, and experiences. This gave them a needed emotional outlet and, by requiring students to reflect on these entries, facilitated an awareness practice on how external settings affect their internal states. Students had one undirected visit with the setting, taking notice of their observations and internal experience.

Post-intervention, students were asked to synthesize their nature observation experience by reviewing and reflecting on their journal entries (2–4 pages, plus images). They were given several prompts that involved analysis of their own entries, such as feelings in the setting; course material influence; urban/nature contrasts; changes in environment and self over time; undirected and directed attention contrasts; and impact of repeated visits over time. They were also asked to return to original definition of environment in their field book and reflect on how their experience affected the way they presently define and conceive of the environment (and, if relevant, other people and oneself). Last, they were asked to reflect on the overall experience and then comment on how (if relevant) the field activities influenced their COVID-19 lockdown experience.

### Evaluation of outcomes

The Reflection Assignment was the central data source for evaluating intervention efficacy based on self-reported experiences and mindsets. Complexity comprehension was observed in definitions and conceptions of environment (pre- and-post-intervention). Standard course assignments were also used verify complexity comprehension: class discussions, an Ecohealth Report, and a Research Proposal. The latter two assignments require students to explore a specific environmental science topic while situating it in sociocultural and economic domains of complex sustainability challenges, including concept maps.

The intervention was not a prepared experiment. It was triggered by a lockdown with uncertain and changing limitations. My decision to use assignments as data came retroactively when I observed the efficacy of this strategy among my students and saw how clearly the outcomes aligned with my theoretical predictions.

I used thematic analysis (Clarke et al., 2015) to deductively identify and code concepts, ideas, and terminology that reflect my objectives and working theory. I read the assignments repeatedly, each time revealing nuances to predetermined concepts and remained open to related yet unexpected themes. Given the storied nature of student personal reflections, I then viewed each as whole narratives of personal experiences, as in narrative analysis (Glover, 2013; Grimwood et al., 2018), seeing causal relationships between themes and participants, and inferring deeper meaning. I tracked thematic relationships using concept maps. I categorized themes (Results) as dominant patterns in the student experience; I include quotations in attempt to reflect lived experiences and interconnected themes, from which the reader may relate to or make further inferences.

I chose not to use participant names or labels to maintain anonymity of personal statements. Statements presented during class and in the Assignment could allow peers to link familiar concepts with personal reflections intended only for my eyes; the absence of labels decreased the likelihood of identification in a small course. Multiple excerpts were taken from each participant (*n* = 15) to reflect different perspectives and experiences.

## Results

The results suggest that this pedagogical method supported student coping, comprehension, and sustainability mindsets during the pandemic lockdown. This is consistent with student sentiments made in class and at the end of the course. Coding uncovered major themes corresponding with their conception and connection to the environment, social connectedness, mental wellness, and a sense of security and stimulation promoted by purpose-directed attention (below), as well as indicators of course material comprehension and application, and the unprompted theme of time perception.

**Figure 1:**
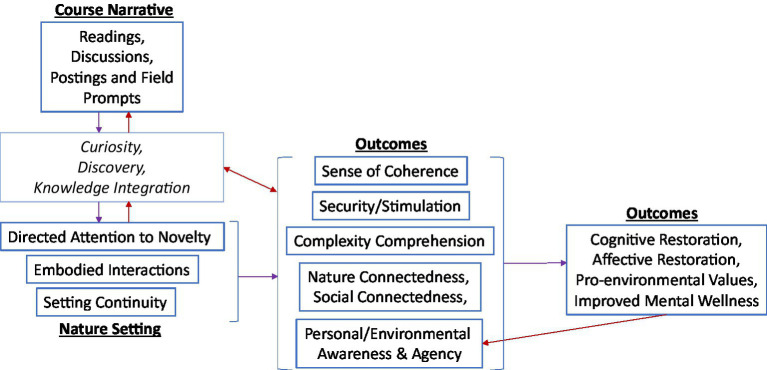
Self-reported outcomes of embodied and purpose-directed attention to a nature setting.

Overall, student testimonies supported my prediction that sense of security and stimulation was moderated by “purposeful” and (semi-)directed attention that became increasingly restorative with meaningful discovery and connectedness (Figure 1). Visits to familiar nature settings and trees (field sites) fostered a sense of security and emerging connection to nature. Course readings, discussions, and online posts prompted a positive anticipation to discover, helped students integrate observations, and enhanced their social connectedness. Students reported improved mental wellness as well as cognitive and affective restoration (immediately post nature). Embodied interactions during directed attention enhanced sense of agency, awareness, and coherence. Students developed stronger pro-environmental value and understanding of the environment as an adaptive complex system. Moreover, some students experienced an identity shift indicative of greater trait resilience: seeing self as part of nature and thus internalizing nature’s adaptive, dynamic complexity. The following qualitative results include further inferences that will be expanded up on with other literature in the Discussion.

### Environment: Concept and complexity

Students initially described *environment* (Week 1) as being relatively “stationary” or “fixed” with “easily defined boundaries.” With recurring nature observations, they came to view the *natural environment* as “multiscaled,” “interactive,” “unbounded,” and “dynamic” “parts that make up a whole.” Several students noted greater “complexity” over time, which they claim would not be visible in one single visit. A student initially saw the environment “aesthetically,” but by Week 3 they were paying attention to “patterns, clusters and arrangements.” When comparing the built and natural settings, the former was characterized by “monotony,” “uniformity,” “manicured,” and “conformity,” and the latter with “diversity,” “uniqueness,” and “equilibrium.”

Scientific and technical prompts initiated “intimate experiences” that led students to see trees as living beings that interact with their environment. This notion that their space was “alive” and “interactive” was often associated with phenological transformations, plant–animal interactions, and student-environment interactions. Students developed the sense that our environment is not a passage, but a living community that is responsive to us—that “humans can also communicate with nature.” This interactive concept came full circle with the idea that the environment is “something we internalize,” that we “can influence and be influenced by.” Students claim the “hands on component” brought this interactive, living environment into a felt experience. It allowed them to see the “placement of plants in space” and watch them respond to weather over time. By digging through the layers of soil next to their tree, they better understood the ecological composition of their landscape and the patterns of relationship between these entities; they read about ecology in course materials, but live observations of one space allowed them to witness “an ecosystem in action.”

Students also developed a more “multifaceted” perspective on what is included in our conception of *environment.* Original definitions were often constructed around a “wilderness” separate from their day-to-day and their own identities. Over time, this grew to include biotic and abiotic factors, built features, humans, and oneself. One student felt that urban settings “deceive us into perceiving environment as a separate entity.” Time with their nature setting strengthened the sense that they are part of the “Earth” or “nature.”

As a result, students’ perception of humanity’s position in relation to the environment also changed. For example, a student saw humanity as one small part of a bigger world that we are not in control of. Students left the course feeling nature was now more accessible, for example, including their own front yard. With the realization that nature “is always there,” we do not need “a getaway,” rather we can simply choose to connect with elements of the environment we are “present” with. When one student came to notice our lack of control over the natural environment, they had reduced anxieties around uncertainty. Ongoing observation of familiar, universal patterns of nature (discussed later in this section) gave a sense of consistency in the world, despite the fact that nature is changing and uncontrollable; this provided better sense of what they “can and cannot change.”

### Environment: Value and connection

With the perception of a “dynamic living” environment and humans as part of that environment, continuous visits nurtured intimate, familiar relationships: “I am part of the living system my tree is in.” This was particularly powerful during a time of social isolation and indoor technological interfaces. One student stated that “paying attention to an individual tree actually created a sense of community.” As their tree changed, they started to pay attention to “phenological rhythm” of other trees, who then became “recognizable characters” around the tree.

The ensuing “personal connection” to nature meant they no longer felt entitled to “exploit” or “profit” from it. In the second semester, one student expressed grief that their trees would be cut down: “I had observed the trees for almost 5 months and [reconnected with them since childhood]. They felt as much a part of me as the veins in my body.”

A greater awareness of their surroundings was accompanied by awareness of our environmental impact and more “eco-conscious” behaviors. They watched the ground being shaped by travel, the movement of litter, and the management of plants. Along with a self-construct embedded in the natural world, this felt sense led to the idea that everyday actions are meaningful and, for at least one student, inspired changes in their everyday habits. In a similar case, it inspired a sense of urgency toward environmental stewardship that paralleled optimism and greater tolerance for ambiguity—the student began to see the “lockdown” constraints as temporary and, relatively speaking, that our “planet is not.” As described above, while students consistently came to see the natural world as uncontrollable, they simultaneously saw human interaction with the environment as influential and meaningful.

All students felt the outdoor activity gave them a “new found” love of nature, also referred to as an “awakening” or being “reborn.” By experiencing and reflecting on their psychological restoration in nature, students claimed to be more aware of nature’s value to human psyche, and thus realized “what is lost when we live without it.”

### Security and stimulation

Habitual visits to familiar nature settings and trees fostered a sense of security: a “constant though the chaos of covid.” Students described their space with terms such as “stability,” “consistency,” “welcoming,” “comforting,” “familiar,” “haven” or “my space,” and feelings like “whole and safe.” The familiarity was afforded by space continuity and emerging sense of connection. Students developed curiosity about their space which sparked a needed sense of “wonder” and “creativity.” They visited it in their free time to exercise or look for new discoveries. By the second semester, some students were driven to independently explore other natural areas near their home and became more familiar with their city.

Course readings and independent research prompted curiosity that could be “satiated” through field activities. For one student, “the more I learned, the more I wondered” and as a result, “my tree gained importance.” For other students, course teachings led to more “appreciation” of these settings, and in turn, a “gradual process” of connection to nature made the course readings “more personal.”

For several students, initial field observations felt “felt like a chore,” a “tedious process,” or distraction from an “immense workload.” One student, who had watched this “mundane” natural setting over several years, likened this experience to piano lessons: by being “motivated to do it externally, it eventually became a treasured part of my life routine…something I looked forward to doing, a reprieve from the monotonous rigor of everything else in my life.” Another student also found the practice “quite mundane in that I had seen the same tree for about 10 years.” With “prolonged periods of time…monotony turned to intrigue” because the “scientific knowledge” of forest ecosystems, invasive species, and environmental management became “remarkably interesting when I was able to point out new aspects of my tree and understand them.”

All students expressed a positive anticipation for arriving in their space—“eager, keen, and excited to visit the same spot each week”—emotions remarkably absent from online learning and lockdown inconsistencies. They enjoyed awaiting birdsong, seasonal shifts, and returning animals. For some, this was carried forward as a generally positive outlook for the day ahead.

Although the prompts and nature itself were changing, nature became something fundamentally reliable: it was “comforting to know that nature often works in similar and predictable ways.” Universal sense experiences of a breeze, smell of leaves after rain, or birdsong, were expressed as a constant over time and place. This was most appreciated by international students isolated on campus away from home: “I took solace in what remained unchanged…there were moments among the trees that I was separate from an unpredictable urban life and I could be certain about things.”

The built environment had the opposite effect. Sounds were “constant but unpredictable” and sense of imagination and comfort were lost “due to calculated structure.” This paradox was apparent in Covid-related anxieties—feeling “exposed” and yet “claustrophobic.” In contrast, outdoor visits became a symbol of safety and freedom: protection among the trees, and yet the ability to discover “without restriction.” In other words, “complexity and perpetuity of nature’s cycles inspired me as much as it calmed me.”

### Purpose-directed attention

Student testimonies supported my prediction that this sense of security and stimulation was moderated by “purposeful” and (semi-)directed attention that became increasingly interesting and restorative with meaningful discovery. Students described their attention as more “specific,” “scientific and “nuanced” over time, as if viewing the world in “higher resolution.” Spotting “new” and “small details” each visit was supported by “encountering the same space every week.” It stimulated an appreciation of nature’s “complexity” and a sense of satisfaction from discovery. Attention to detail was, by student accounts, afforded by “purposeful” course material and directed attentional prompts. Referring to phenology, a student said, “I learned to pay attention because of the course material.” For another, “it was only after reading about soil in our textbook that I could make in-depth observations.” The impact on one student was a shift from focusing on “major cumulative changes” (like seasonal transitions of a forest) to noticing “smaller changes in individual trees.”

Students made subtle discoveries in highly urbanized settings. Feeling stunted in a relatively sterile urban yard of Dubai, a student was inspired by learning that trees increase biodiversity acting as habitat and food; they decided to “pay more attention…notic[ing] many insects such as ants over the bark…and cicadas that formed a constant background noise…*Instead of a single tree, I saw it as a bustling microcosm full of life*.” Another student similarly came to see their tree as “home to the living organisms in its personal ecosystem, such as the wasps’ nest perched on its branch and the insects residing at its roots.”

Appreciation for detail and emerging curiosity was afforded by embodied experiential learning, including their ability to enter spaces, interact with them, and look at living things from “different angles” than they normally would. Students reported enhanced sensory awareness to aspects of the natural environment that typically go “unnoticed.” They explicitly noticed that engaging with the environment helps us be more aware of it: “to feel the texture of the dry and hard leaf…smell the dirt in which it landed in with the wind…we make contact…we develop feelings for it. We love it so much.”

Beyond the course, students carried an ongoing “general awareness” of their surroundings and how they are internalized. After the first term, one student began to “make a conscious effort to take notice when I am in nature. I am aware of my senses as I watch the birds fly overhead, feel the wind and rain on my face, and listen to the rustle of squirrels in the trees.” Students claim that attentional prompts and journal reflections gave rise to a “more conscious effort to be aware” of these interactions. A student gave the example of going into new settings being more aware of not only visible plant life, but also feeling “mobile forces such as the wind against my skin and how it could be carrying seeds and insects. The stationary, fixed environment I once appreciated in lifeless photos has now bred life and movement with bounds undefined.”

Students independently identified benchmarks to measure subtle changes in their settings over time. For example, one student used a rock as an indicator of changing water levels in a creek. Setting continuity provided recurring features as markers of ecosystem changes and interactions, supporting field observation skills that are not easily established in a fast-paced, technocentric world. In fact, students felt that being away from technology in and of itself was a major factor permitting more attention to their surroundings and establishing this as a life skill.

While student observations increased in specificity, they did so without losing attention to the community as a whole. As shown above, students had greater sensitivity to interactions between entities that made up their surroundings. Overall, they gave more attention to subtle changes over time without isolating entities of the environment from meaningful relationships. One student described paying new attention to details of leaf shape and bark texture, then “over time analyzing the environment surrounding my tree and how that would affect its development,” and then extending this to surrounding urban development.

During the undirected attentional prompt, students reported that they initially focused on features from previous activities. Eventually, students let their mind “wander,” extending observations to other features and interactions. One student reported more attention to the “overall environment” when left undirected. Another felt “lost yet bonded to nature.”

### Social connection

Despite extremely limited opportunities for social exchange, this approach seemed to have a positive impact on student social relations and sense of social connectedness during the lockdown. They bonded over a unique experience and sharing stories through common experiences, disciplinary vocabulary, and narratives of the course.

Weekly activities influenced social relations outside of our group; sharing discoveries with family members, or feeling connected to ancestors associated with the space. For some students isolated on campus, flora and fauna replaced a social community. They commonly spoke about their tree in a personified manner, and some made regular visit to their “old friend.”

Students made interesting observations of social interactions in built and natural settings that enhanced their appreciation of nature as a social moderator. They noted a greater sense of community and connectedness among strangers in public nature settings (like parks) in comparison to the “colder” social structures of built settings. With “eyes drawn downward,” urban environments were described as channels of transportation that “have almost made us as humans lonelier and more disconnected.” Even when people were not engaging directly in nature, they shared joint attention “engaging in the same beautiful scenery together.”

Students themselves felt it was more socially acceptable to pause, observe and socialize when in nature. In the built environment, “I felt uncomfortable in my own skin, as if I had to be busy or look occupied to other people. Yet in my nature spot I felt a sense of release.” The student believed urban settings favored “progress and power,” while nature provides an “organic sense of community and connectedness…that is so comforting…we are reminded that we are all a part of Earth.”

### Mental wellness

Mental wellness benefits came in the form of stress reduction, cognitive restoration, optimism, trait resiliency, self-discovery, and “healing.” While some of these outcomes have been revealed above, I will present others, draw attention to moderators of these outcomes, and share student awareness and interpretations of these outcomes.

Mental wellness impacts of lockdown were expressed as feeling “confined and alone,” “hopeless,” “limited,” “lonely,” and “detached from reality,” along with the “unhealthy patterns” of behavior. One student expressed it as “a collective experience that feels fundamentally singular…a societal grief with no reprieve.”

Students consistently reported feeling less stressed after their visits, using terms such as “less anxious,” “more relaxed,” “joyful,” “happy,” “tranquil,” “passion,” “calmer,” “warmer,” “lighter,” “healthier,” and “serenity.” They felt a sense of “companionship,” “comfort,” and “passion” absent from online learning. One student shared that, “going to my nature spot gave me a sense of control in my life when it felt like I had no control over any circumstances;” another felt “truly at peace” in the setting despite “uncertain times.” Even when away from this setting, students “took comfort” in knowing they could return to nature when feeling “burned out” or “overwhelmed.” Another found “peace of mind that no other place gave me.”

Reflections also indicate cumulative and enduring impacts on student wellness over the course of the nature observations: “through this experience I have been able to find therapy and serenity through nature…in the past few months alone, my anxiety and other mental health issues have decreased.”

It was common for students to feel heightened anxieties and discomfort during initial visits. For example, one student felt “anxious about the work I am putting on hold,” but this dissipated once they started to “focus on the task at hand.” Reflecting on weekly journal entries, one student noticed “unhealthy patterns of thought that began to shift,” and another that their “tone became more positive.” Learning about the natural world gave a student “a sense of purpose.” Another student felt nature itself can “teach us joy and comfort.” Nature’s behavior taught a student how to not be “overwhelmed” and another how to adapt to disturbance.

Therapeutic effects were often phenomenologically based; meaning, the ability to interact with the setting was fundamental to coping outcomes. A student expressed that the pandemic isolation left them “trapped with my inner monologue”; nature observations helped this person “listen to what I could feel rather than my own thoughts. To embrace something external and receive new input.” Feeling “connected” to the world, it “reignited an enthusiasm to be explorative and creative.” Despite the scientific nature of most activities, an enhanced embodied awareness commonly followed experiences of positive emotions in their nature setting. Students communicated the disembodying effect of online, isolated learning with comments like: “the discourse between my mind and my body began to stagnate.” Nature-based observations acted as a “grounding mechanism” that made them feel more “connected to reality” through hands-on activity in a time when “everything in my world was at a distance.” For some, this was the ability to simply “breathe deeply again,” “listen to what I feel,” or tap into the “organic sense of community and connectedness.”

The awareness that we internalize our environment was realized through embodied experiences; students became “more aware of the healing power of nature” by spending “more time outside engaging with nature.” They noticed how they internalized the sound of calm water or the relaxation that came from a “serene view.” Recall simple sensory awareness, like the unsettling feeling from constant, unpredictable sounds of urbanicity. One student detailed how people in the built space seemed to be “reproducing aspects of [their] external environment. Like the gray rows above and the grids on the ground, their behavior was characterized by order, conformity and efficiency.” Recall how built spaces made students feel the need to be busy and isolated; nature gave permission to be still, observe and engage with others.

Students experienced shifts in self-awareness, self-discovery, and identity. The impact of lockdown isolation, for one student, was a sense of “missing part of myself.” A “nostalgic” desire to rebuild connection to nature once held in childhood was common, and it took form of a desire to reconnect with a past self. An unexpected outcome of the nature-immersion activities was that it helped some students “discover who I am,” expressed also as a “sense of place,” “peace, confidence and intimacy” with oneself. This self-discovery, as noted above in environmental constructs, emerged from a belief that we are part of nature and dependent on it: “it was in studying the intricacies of its branches, soil, and leaves over time that felt as if I discovered more about the features that make me who I am.

The self as part of nature had a “humbling effect,” with a shift in perspective that we are but “one small part of a complex world.” In turn, anxiety-inducing problems lost their significance. Connecting “with something greater” was both “humbling and exciting,” and for some, fulfilled “spiritual needs.” A spiritual “healing experience” was particular powerful for those who identify as “non-religious.” A student in “desperate need of stability and healing” believed their tree brought healing through the ancestor who planted it: “ironically enough, as the leaves turned brittle and fell to the ground, my body and mind healed. It was almost as if the tree had given me its lifeforce as it entered its state of dormancy.”

Awareness of nature’s restorative capacities seemed to help students stay present with the activities, losing the idea that they were distracted from studying, and concluding that “time with nature is never time wasted.” Students became increasingly aware of their enhanced cognitive performance after visiting their nature setting and, in turn, took away important lessons for balanced study practices. Further, time for “introspection” during nature visits or journal writing gave space for reflecting on who they are and how that aligns with how they spend their time and what they give their attention to. For example, “it was made clear to me that it was what you choose to focus on that can change, and it might change you.” For some, this awareness encouraged healthier behaviors, as they found themselves exercising more and taking initiative to get outdoors.

### Course goals and application

Consistent with previous years, students developed a greater capacity to make hypotheses about ecological interactions and causal relationships, and to think critically about complex socio-ecological systems. Assignments demonstrated the ability to apply observations of interconnectedness to interdisciplinary dimensions of sustainability. Reflection Assignments suggest that students were aware of this lesson, stating for example, that “as this course has progressed, my scientific knowledge of Earth’s elements, anthropogenic influences, and multidisciplinary approaches…increased tremendously, [including] the interweaving of Earth science and political, social, and economic justice, with the goal of equality and sustainability.”

Students reported benefits of science accessibility in applied settings with “kinesthetic and visual” learners being able to “touch and feel” at their own pace. A student stated: “I assumed I was someone who could never do science [but found] a new passion in the sciences that I could have never predicted.” Students typically leave this course with greater curiosity about how the natural world works. Anecdotally, this year’s cohort was extraordinary in their sense of nature connectedness, nature curiosity, and capacity to understand complexity.

### Time perception

A recurring, unintended theme was a shift in sense of time. Several students noted they were initially unaware of how online learning was distorting their sense of time until they started working with the nature setting. A student described, “leaving technology behind when I visited my tree made time pass only as quickly as I felt it” and associated this with attention to embodied sensory experiences in the moment and reduced anxieties about the future – in turn, it restored their sense of “subjectivity” and they felt “more in control.” Others became more aware of how fast nature could change, or how long it takes for leaves and acorns to fall.

## Discussion

According to my students, nature-directed learning during the lockdown equipped them with a greater sense of connectedness and wellbeing, as well as sustainability mindsets, behaviors, and capacities. The preliminary results herein suggest these outcomes were supported by synergistic feedbacks between nature-immersion activities, course readings, discussions, assignments, and reflections.

Embodied engagement with nature played a significant role in the desired outcomes of this intervention – this is no surprise, given what we already know academically and intuitively about online learning and nature-immersion. The unique aspects of this work that I want to emphasize in this Discussion are the role of *curriculum*—readings, discussions, assignments, inquiry/prompts, reflections, and overall narrative—and *setting continuity*. First, they have to capacity to support meaningful dialog between sensory/perceptual and conceptual/theoretical processing, which has outcomes on awareness, sense of coherence, and personal and environmental agency. The way nature is presented in the curriculum narrative thus has an important role in shaping what is internalized during this dialog. Second, they provide a needed freedom and structure, which, in conjunction with purpose-directed attention to nature, may enhance cognitive restoration, meaningfulness and sense of connection. Last, I highlight the role of collective and reflective knowledge integration in this process, particularly in sustainability-situated sciences, which can support profound shifts in how students perceive themselves, others, and the environment.

Scholars of “Place-Based” outdoor pedagogies examine the lived, embodied experiences that emerge from an *ongoing relationship* to a particular setting. They essentially apply the concept of place attachment—a bond formed between individuals and meaningful settings (Scannell and Gifford, 2010:1)—to pedagogy. Similar to my findings, place-based outdoor learning has helped youth apply knowledge to real-life challenges and understand social, cultural, economic, and physical interconnectedness in environmental decision making (Nielsen, 2016). Further, the “attentive connection” has a formative impact on sustainability identities, including greater care for the natural and human elements (Bates et al., 2019: 95). Attachment and meaning emerge over time as students “re-engage with “the active, perceiving, and sensuous corporeality of the human experience,” also referred to as “ecocentric intercorporeality” (Payne and Wattchow, 2009: 16). Interpretations of the body, mind, and setting are harmonized into one coherent meaning-making reality. This emerging sense of coherent reality was common among my students.

Sensory-cognitive-setting connections were muted by lockdown learning, sitting in a chair, occupying the mind with abstract concepts unrelated to settings and sensations of their present realities. The associated distortion in sense of time expressed by my students is a common pandemic sentiment. We lack the ability to plan, maintain routine or experience normal temporal benchmarks of seasonal or annual gatherings, and we are isolated from people and places as they change. One study showed that, compared to urban settings, walking in nature slowed time perception and was accompanied by stress reduction and improved mood among university students (Davydenko and Peetz, 2017).

Payne and Wattchow (2009) suggest that such shifts in perceptions of time and space in place-based learning are facilitated by uniting of our sensory-perceptual and conceptual-theoretical processing. In other words, we are in “real time” when the theoretical concepts we are thinking about are enacted through our present embodied sensory experiences drawn from the present environmental setting. Nature activities restored sense of time, reality and awareness for my students as their mind and their body attended to the same environmental stimuli in a given moment. By paying attention to details and relationships of these living stimuli over several weeks, they could witness subtle changes in relation to time. Further, the meaning of sensory-stimuli interactions was enriched by these changes and course theory over time.

Based on this understanding, I want to highlight the importance of the conceptual course narrative in shaping how students conceive of nature, and thus, how they interpret their embodied nature observations and integrate them into meaning-making realities. Student sense of hopelessness, uncertainty, and lack of control subsided over the course; collectively, they related this not only to sensory awareness and connectedness, but also ongoing discovery of nature as interactive, dynamic, complex, self-inclusive, and responsive to their actions.

Our conceptual framework of nature, constructed in a given sociocultural context, is a lesser studied yet likely influential factor in the dynamics of nature connectedness, pro-environmental behavior, and psychological wellness. The framework for understanding environmental science and sustainability in this course was adaptive complex systems thinking: living entities interact, self-organize, and adapt to give rise to non-linear and unpredictable dynamics of evolution.

Leong et al. (2014) found that people with higher nature connectedness also tend to be more innovative and holistic thinkers, as measured by the Analysis-Holism Scale. The scale uses indicators of complexity reflected in student testimonies with statements like “the whole is greater than the sum of its parts.” Recall how Mirella Stroink (see Introduction) has shown the compatibility of systems thinking with all the traits of sustainability mindsets as presented herein.

According to both Indigenous and Western scholars, complexity of nature is more easily understood when we are immersed in it and see ourselves as part of nature (Davis and Sumara, 2000; Salmón, 2000; Du Plessis and Brandon, 2015). Indigenous knowledges emphasize that this awareness is both phenomenologically and culturally constructed (i.e., land-based activities and stories) and helps us understand that humans are subject to the same principles of interconnectedness and adaptation (Salmón, 2000; Nabigon and Wenger-Nabigon, 2012).

My students’ testimonies of personal growth and mental wellness in response to the intervention are collectively represented by *trait resilience—*the cognitive and emotional flexibility that allow us to adapt to changing situations (Hinton and Kirmayer, 2017). With a conceptual narrative of complexity priming nature-immersions, when my students witnessed, related to and connected to nature over time, their self-discovery was more likely to take on resiliency traits of adaptive systems. Students felt they internalized traits of nature and learned from its behavior. By viewing the environment as a dynamic and permeable space we are all part of, they felt less lonely, discovered that we cannot control the world, and yet also realized their interactions with the environment are impactful and meaningful.

Attachment to place is influenced by individual and group identities that form our understanding of that place and the culturally meaningful activities we do there (Low, 1992; Bonaiuto et al., 2019). In turn, the culturally meaningful symbols of place are internalized as they shape our thought patterns and identity. Thus, setting activities and conceptual theory can be structured to empower students with a dynamic, adaptable, and process-oriented concept of nature that is both externally and internally applied.

Another outcome of a self-construct embedded in nature is the long held Indigenous understanding that the wellbeing of humans and nature are synonymous, and an associated environmental concern (Adelson, 2000; Nabigon and Wenger-Nabigon, 2012). My students became more aware of how we internalize the environment physically, psychologically, and, for some, spiritually. Other research shows that people tend to go to their favorite settings for restoration and emotional self-regulation—which are most often natural settings with beautiful scenery, complexity, and richness—over time, these settings form part of their identity (Korpela and Hartig, 1996; Korpela et al., 2020; Folmer et al., 2013). With the concept an interactive environment, my students were empowered with a conscious ability to self-regulate their emotions by changing their settings as well as their focus of attention. They took comfort in knowing they have this capacity.

Thus far, we see how conceptual and perceptual experiences of the intervention provided students with a greater *sense of coherence*, where internal and external stimuli are understandable, manageable, and meaningful (Antonovsky, 1996; Eriksson and Mittelmark, 2017). As a result, students were motivated to be agents in the environmental movement as well as their own internal being.

Outdoor adventure recreation for university students involves autonomous learning through physical and mental challenges that not only enhance mental wellbeing and nature connectedness (Kanters et al., 2002) but also sustainability mindsets of agency, resilience, and willingness to take risks (Takayama et al., 2018; Davidson and Ewert, 2020). While the intervention did not involve physical challenges, it provided ample other opportunities for agency-building through autonomous learning and problem solving. Students chose their field sites and assignment topics and learned at their own pace in their own time. With each visit they discovered new input, directed neither by their own habitual patterns of thought nor prefabricated information being fed through a computer screen, but through exploration, creativity and watching life unfold.

Autonomy in how and what they learn gave students a sense of freedom absent from online learning. In another case of integrated outdoor learning, Grimwood et al. (2018) found that curiosity, intrinsic motivation, and sense of autonomy are inspired by providing more questions than answers and directing attention toward patterns in the environment rather than labeling components of the environment. They used a range of techniques like ceremonial routines, arts, introspection, wandering, challenges, and exploration.

Along with freedom, student experiences in my course were also imbued with security, stability, and non-randomness. With the assumption that social, physical, and work life are resource depleting, we see an abundance of studies measuring psychological restoration during passive experiences like walking, sitting, or meditating in unfamiliar natural settings. The intervention provided more structure and depth of connection to setting than passive mind wandering.

Scientific inquiry and applications directed attention to new patterns, detail and relationships in the same setting that progressively gained value and meaning over time. Students learned to pay attention and stay present with their senses, but were eventually motivated by their own curiosity and effortless fascination.

Williams et al.' (2018) theory of creative thinking during psychological restoration in nature proposes that internally oriented “mind wandering,” which offers flexible thought, is alternated with externally oriented soft fascination with environmental stimuli, which grounds these thoughts with structure. Consistent with this, attention during undirected observations of my intervention was described as free and wandering, yet bonded to nature. However, this took place after weeks of learning to pay attention and connecting to nature.

Most ART studies to-date measure directed attention capacity after undirected nature experiences. Research in nature-based mindfulness demonstrates that semi-directed attention to nature itself seems to enhance effortlessness and restorative benefits and, further, that awareness of the present can be learned by directing attention to nature. The initial stages of learning mindfulness meditation are effortful and actually deplete cognitive resources. Yet this effort is reduced when the practice is directed at natural scenery, and thus seems to be moderated by interest, pleasure, and curiosity (Lymeus et al., 2017). Directed attention toward specific elements of nature (e.g., leaf color, smell of air) can not only enhance mindfulness during a walking mediation, it can enhance mood and nature connectedness (Nisbet et al., 2019). Mindfulness practices are a form of attending to the present moment by drawing attention and awareness to sensory experiences (Hinton and Kirmayer, 2017). As expressed by my students, this can reduce ruminative and self-critical thought patterns.

Thus far, restoration research on directed attention to elements of nature does not consider their sociocultural meaningfulness, reflecting another underlying assumption of Kaplan and Ulrich that is overemphasized: restoration is mediated by fascination with nature’s beauty. When nature is removed from our purposeful daily routines, we become spectators who only see its beauty, leading to very different outcomes in our human position and possibilities. Recall the student who initially saw the environment “aesthetically.” With directed prompts and scientific understanding, they began paying attention to “patterns, clusters and arrangements” and saw the environment as increasingly interactive, complex and part of our living community.

The overall course narrative facilitated understanding of nature observations and provided meaningful vocabulary that supported both theory comprehension and a shared language for communicating these experiences in a social community. As stated by Sato and Conner (2013), the *how* of paying attention to nature matters, and we can actively cultivate our fascination and wonder in nature. This is significant to sustainability pedagogies in world no longer directly dependent on navigating natural patterns or associating these symbols shared cultural meaning.

Over the term, the students seemed to experience a cycle of security, stimulation, curiosity, and connection with nature. I want to emphasize that setting continuity and purpose-directed attention appear to be key factors in the level of familiarity and curiosity in this cycle. Attention that would otherwise by occupied by a high degree of novelty was directed at discovering finer details previously unnoticed. Unlike lockdown restrictions, novelty in their nature settings involved subtle changes that were relatively easy to integrate with nature conceptions and course narratives. Thus, the ability to witness recurring patterns in both nature and sensory experiences revealed a permeance in natural laws that students could be certain about, yet a dynamism that kept them wondering. This was aptly summarized as: “the complexity and perpetuity of nature’s cycles inspired me as much as it calmed me.”

Students made numerous statements like this, which I believe reflect Kaplan’s Preference Matrix based on understanding and exploring the environment. For example, they were able to draw upon familiar, reliable orientation points in their setting to help them measure novel interactions of a dynamic living system. Further, the course sparked a sense of wonder and curiosity that drove a desire to understand the meaning of these interactions. While the Matrix describes why certain settings are preferred, the present work demonstrates how we can encourage understanding and exploration with setting recursiveness and purposeful inquiry. Students did not move to a preferred setting, rather their *perception* of the setting itself had shifted.

Students initially experienced anxieties, disinterest, and tediousness in a mundane setting, traits they also used to describe the lockdown experience (monotonous/unpredictable). Their newfound love of nature as comforting and engaging was accompanied by a new sense of self. Others have found that personal growth in the form of self-discovery and self-efficacy in outdoor learning initially involves a challenging period of discomfort and negative emotions, followed by a period of transformation as one overcomes these challenges (Quay et al., 2002; Taniguchi, 2004). Not only are they left satisfied with a sense of accomplishment, the new self holds greater trait resilience and capacity to undergo more growth in the face of new challenges (Hinton and Kirmayer, 2017). As alluded to by my students, the ability to process this cognitive and identity restructuring is supported by reflective writing and collective discussion (Taniguchi, 2004). We see something similar in awe experiences inspired by beauty, novelty, and complexity of natural phenomena. Keltner and Haidt (2003) propose awe starts with wonder and is followed by discomfort as cognitive structures adjust to making sense of the novelty. If this newness is well integrated into meaningfulness, it can end in profound personal growth. In sum, novelty stimulates growth and resilience when it is well integrated into a stable state.

Knowledge integration and reflection in my class were facilitated by unrestricted nature visits, field book observations, personal journaling, and class discussions, all culminating in the Reflection Assignment. Periods of introspection were associated with greater self-awareness and sensory awareness. The opportunity to look back at their field book entries with the Reflection Assignment made students aware of how their cognitive and emotional states changed over time—and in response to different conditions—and to further integrate observed novelties into a coherent narrative. Importantly, the common discourse and shared sense of purpose realized during class discussions provided social connection in lonely time.

Straker (2014) offered a similar multi-dimensional approach, observing that outdoor educators inspired meaningful engagement and respectful relationships by encouraging pro-environmental values in applied settings, opportunities for overcoming challenges, spontaneous learning, and quiet introspection. Sharing outdoor discoveries among a social community has shown to strengthen connection to nature (Grimwood et al., 2018). Journaling, gratitude practices, and meditation in a nature expedition supported both social and ecological care, referred to by Nielsen (2016:9) as an “ecology of giving.” During an introductory environmental studies class, weekly multimedia nature journals (i.e., Blogs) increased nature awareness and outdoor interest, without outdoor exposure (Arnold, 2012). Like my students’ testimonies, these outcomes were mediated by greater awareness of seasonal changes and understanding the social context of the nature setting (the neighborhood).

Narratives of nature-immersion pedagogies have an important role in scientific comprehension, agency, and sustainability applications, as demonstrated by my students’ testimonies and assignment applications. Simple inclusion of natural elements in a university classroom improves knowledge retention (Holden and Mercer, 2014). Science classes situated in applied outdoor settings has been associated with a sense of accomplishment—in the case of nature restoration (Ernst et al., 2006), better performance in science, reading and writing—when studying a wetland (Santelmann et al., 2011), and greater intrinsic motivation, competence, and autonomy—in the case of outdoor adventure (Mackenzie et al., 2018). In all these studies, science was made more interesting and enjoyable.

Kuo et al. (2019) propose that academic performance in nature-immersion pedagogies is moderated by greater interest, stress reduction, cooperation, and sense of security in a “calmer” context, as well as the tendency to offer more autonomous learning in these settings. Almost all of these attributes were presented by my students as moderators (no opportunities for social cooperation in nature). As stated above, scientific knowledge played a major role in engaging student interest and intimacy with their space and shaping interpretations of sensory experiences.

The personal and academic outcomes—notably, sense of coherence, complexity, and agency—also demonstrate the importance of delivering environmental science in a way that does not solely direct attention to discrete and separable details, but also to relationships in a dynamic living setting. Processing scientific discovery in a social setting and applying it to broader social realities seems to improve student interest and influence sustainability mindsets.

## Conclusion

This nature-immersion intervention during a pandemic lockdown provided a consistent learning space that meaningfully engaged student minds and bodies and improved self-reported mental wellness. By the end of the term, students perceived their internal states and nature itself with a greater sense of security and stimulation. They demonstrated sustainability mindsets and wellbeing outcomes that are relevant beyond lockdown scenarios. This pedagogy of freedom and structure empowered sense of coherence, awareness, and agency through ongoing dialog between theory and immersive experience.

This work suggests that directed attention to nature is increasingly effortless when intrinsically motivated by a cultural narrative of meaningful relationships that asks us to derive and apply information drawn from nature. A cycle of meaningfulness, effortlessness, connectedness (nature and social), curiosity, discovery, and restorativeness may be enhanced by recurring visits to the same setting over time, mixed with periods of collective and introspective integration.

By directing attention in this manner, nature-immersion inquiry is an engaging and accessible pedagogical tool that can be used in simple settings (e.g., tree) with many different learning styles that can teach students how to pay attention. We also have the opportunity to be conscious of the underlying constructs of nature we present—a beautiful escape or a meaningful teacher and community—influencing student capacity to see adaptive complexity in sustainability systems and themselves.

This paper challenges the notion that daily routines and real scientific rigor are necessarily resource depleting and apart from affective sensory experiences in nature. We need not assume punitive styles of higher education that require restoration; we can harmonize internal and external meaningfulness to engage and inspire the whole person.

## Limitations and future research

The degree and direction of study outcomes were not measured. However, isolating linear causal effects may not be useful to interdisciplinary, integrated pedagogies. The outcomes were analyzed from a student assignment, which risks influence from impressions of professor expectations. Students documented observations and insights over the term and communicated them without any knowledge of my strategies and working theory. Student perception is the focus of this inquiry, which has provided plenty of insight for pedagogical applications and future research. Notably, the results compel empirical investigation on contexts driving purpose-directed attention, and the outcomes on fascination, attention effort and restoration.

## Data availability statement

The datasets presented in this article are not readily available. Requests to access the datasets should be directed to nicole.spiegelaar@utoronto.ca.

## Ethics statement

The studies involving human participants were reviewed and approved by The University of Toronto’s (U of T) Human Research Ethics Unit (HREU). The patients/participants provided their written informed consent to participate in this study.

## Author contributions

The author confirms being the sole contributor of this work and has approved it for publication.

## Conflict of interest

The author declares that the research was conducted in the absence of any commercial or financial relationships that could be construed as a potential conflict of interest.

## Publisher’s note

All claims expressed in this article are solely those of the authors and do not necessarily represent those of their affiliated organizations, or those of the publisher, the editors and the reviewers. Any product that may be evaluated in this article, or claim that may be made by its manufacturer, is not guaranteed or endorsed by the publisher.

## Footnotes

^1^Kaplan and Kaplan (1989 p. 73, 95) acknowledged that ancestral attention would be influenced by acquiring food, cultural background and sense of belonging, yet these attributes are overshadowed by "getting way" in operational studies.

^2^This course is situated in the Trinity One Butterfield Environment and Sustainability Stream of Trinity College, University of Toronto.
